# Fucoidans as Potential Inhibitors of HIV-1

**DOI:** 10.3390/md11083000

**Published:** 2013-08-19

**Authors:** Maria M. Prokofjeva, Tatyana I. Imbs, Natalya M. Shevchenko, Pavel V. Spirin, Stefan Horn, Boris Fehse, Tatyana N. Zvyagintseva, Vladimir S. Prassolov

**Affiliations:** 1 Laboratory of Cell Biology, Engelhardt-Institute of Molecular Biology, Moscow 119991, Russia; E-Mails: m.prokofjeva@gmail.com (M.M.P.); discipline82@mail.ru (P.V.S.); prassolov45@mail.ru (V.S.P.); 2 Laboratory of Enzyme Chemistry, G.B. Elyakov Pacific Institute of Bioorganic Chemistry, Far Eastern Branch, Russian Academy of Sciences, 159 100-Let Vladivostoku Ave., Vladivostok 690022, Russia; E-Mails: tatyanaimbs@mail.ru (T.I.I.); natalyshe@piboc.dvo.ru (N.M.S.); 3 Research Department of Cell and Gene Therapy, Clinic for Stem Cell Transplantation, UCCH, University Medical Center Hamburg-Eppendorf (UKE), Hamburg D-20246, Germany; E-Mails: shorn@uke.uni-hamburg.de (S.H.); fehse@uke.de (B.F.)

**Keywords:** fucoidan, brown algae, antiviral activity, human immunodeficiency virus type 1

## Abstract

The antiviral activity of different structure fucoidans (α-l-fucans and galactofucans) was studied using two model viral systems based on a lentiviral vectors and a replication competent Moloney murine leukemia virus (Mo-MuLV). It was found that investigated fucoidans have no cytotoxic effects on Jurkat and SC-1cell at the concentration range of 0.001–100 µg/mL. Fucoidans with different efficiency suppressed transduction of Jurkat cell line by pseudo-HIV-1 particles carrying the envelope protein of HIV-1 and infection of SC-1 cells by Mo-MuLV. According to our data, all natural fucoidans can be considered as potential anti-HIV agents regardless of their carbohydrate backbone and degree of sulfating, since their activity is shown at low concentrations (0.001–0.05 µg/mL). High molecular weight fucoidans isolated from *Saccharina cichorioides* (1.3-α-l-fucan), and *S. japonica* (galactofucan) were the most effective inhibitors.

## 1. Introduction

Virus entry into the cell is a key stage in the extension of viral infection. Most enveloped viruses enter the cell only after interaction of viral envelope proteins with the specific receptors on the cell surface. For many viruses it was found that the contact with the specific receptor is preceded by non-specific interaction with the primary receptor. It was shown that heparan sulfates located on the cell membrane are involved in primary cell binding of many viruses (herpes simplex virus, hepatitis C virus, human cytomegalovirus, human papilloma virus, HIV-1, murine leukemia virus) [1]. The contact of retrovirus with the primary receptor is required for subsequent strong interactions with receptor/coreceptor specific for retroviruses of each group of interference [1,2].

Thus, the search for effective inhibitors that inhibit the interaction of viruses with cellular heparan sulfate is an important task, the solution of which can lead to the creation of a broad spectrum preparation effective for different groups of pathogenic retroviruses including HIV-1 [3].

Sulfated polysaccharides are known to demonstrate a high antiviral activity preventing penetration of DNA- and RNA-containing viruses to the cell [4]. Their degree of activity directly depends on the following sulfated polysaccharide properties: molecular mass, structure of carbohydrate backbone, sulfation degree [5]. For some classes of semisynthetic polysaccharide (e.g., dextran sulfate) it is known that their antiviral activity increases while the sulfation degree and/or molecular mass increases too [6,7,8].

Natural algae sulfated polysaccharides demonstrate an anti-HIV activity which is comparable to dextran sulfate activity [5,8].

Fucoidans—sulfated polysaccharides from brown algae—are heterogenic and represent the mixtures of structurally related polysaccharides with certain variations of carbohydrate units (l-fucopyranose and non-fucose ones) and non-carbohydrate substituents (mainly sulfate and acetyl groups) content. The detail structural analysis of fucoidans is difficult at present, so structural variety of fucoidans has not been investigated yet. The literature data analysis shows that most of known fucoidans fall into three structural types: the first type contains (1→3)-linked α-l-fucopyranose residues in the main chain; the second type alternating (1→3)- and (1→4)-linked residues of α-l-fucopyranose; and the third type is fucoidans containing galactose and fucose in comparable quantities (galactofucans). Besides fucose, many fucoidans contain some quantities of other monosaccharides [9,10]. Rarely found are fucoidans with the main chain represented by uronofucans [11,12].

In contrast to semisynthetic sulfated polysaccharides, natural algae polysaccharides do not show any discernible anticoagulant activity at therapeutically administrable doses [7]. Advantages of the algae polysaccharides are their relatively low cost, wide spectrum of antiviral activity, absence of expressed cytotoxic effect, low drug resistance formation degree during long-term use, and good solubility [5].

At present, there are two main approaches to determine the efficiency of potential antiviral agents. The first is *in vitro* inhibitory analysis using purified viral enzymes [13]. However, when working with purified enzymes, it is impossible to determine the ability of potential drugs to interact with the cell *in vivo*, in particular their interaction with cellular receptors, the ability to penetrate into the cell, stability, and the absence of cytotoxicity. The second approach involves working with infectious viruses, such as HIV. This approach can only be applied in a small number of laboratories that are specially equipped for work with human pathogens [14]. Limitations in working with infectious viruses greatly complicate the search for and testing of antiviral drugs. Using the system designed at the Engelhardt Institute of Molecular Biology of the Russian Academy of Sciences, it is possible to investigate the activity of potential inhibitors that prevent the interaction of simple and complex retroviruses with the primary surface receptors of the cell thus preventing the infection of cells [15,16]. The aim of this work was to study the antiviral activity of a number of natural fucoidans and choose the most active fucoidans samples to be studied further as potential candidates for antiviral drug development.

## 2. Results and Discussion

### 2.1. Characterization of the Fucoidans from Brown Algae

The antiviral activities of fucoidans from different species of brown algae isolated from the Far-East Russian coast were investigated. Fucoidans were represented with different structural types (Table 1). The polysaccharide chain of the α-l-fucan (ScF) isolated from *Saccharina cichorioides* was built up mainly of (1→3)-linked α-l-fucopyranose residues. Other α-l-fucan (FeF) isolated from Fucus evanescens contained the backbone with alternating (1→3) and (1→4) linkages. The galactofucans (GF) SgGF, AoGF, SjGF were obtained from brown algae *Saccharina gurjanovae*, *Alaria ochotensis*, *Saccharina japonica*, respectively. 

**Table 1:** Characteristics of polysaccharides fractions from brown algae.

Seaweed source	Fraction	Mw, kDa	The linkage between Fuc residues	SO_3_Na^−^ (%) *	Monosaccharide composition, mol%
					Fuc/Gal/Man/Rha/Xyl/Glc ***
*α-l-Fucans (F)*					
*S. cichorioides*	ScF	1160	α-1,3	26.5	88.6/6.4/3.1/0/1.9/0
*F. evanescens*	FeF **	620	α-1,3;1,4	23.2	77.9/9.5/4.2/0/8.4/0
*F. evanescens*	FeFDA	20	α-1,3;1,4	21.3	82.7/8.7/3.0/0/5.6/0
*Galactofucans (GF)*					
*S. gurjanovae*	SgGF **	810	α-1,3	28.2	64.3/20.7/14.6/0.4/0/0
*Al. ochotensis*	AoGF	860	α-1,3	24.0	53.8/38.5/7.7/0/0/0
*S. japonica*	SjGF **	1800	α-1,3;1,4	23.3	49.9/44.1/5.3/0/1.1/0
*C. costata*	CcGF **	160	α-1,3	23.2	62.6/30.1/2.6/1.7/0/0

* % of sample weight; ** acetated sample; *** Monosaccharide composition: Fuc = fucose, Gal = galactose, Man = mannose, Rha = ramnose, Xyl = xylose, Glc = glucose, SO3Na^−^ = sulfate group.

All investigated α-l-fucans and galactofucans were highly sulfated. Gel-permeation chromatography investigation of fucoidan samples demonstrated their comparable elution profile and domination of the polysaccharide fractions with molecular weights (Mw) ranged from 300 to 800 kDa. The fucoidans with Mw higher than 1000 kDa were isolated from *S. cichorioides*, *S. japonica*. According to our recent studies, the α-l-fucan from *F. evanescens* and the galactofucans from *L. gurjanovae*, *C. costata* and *S. japonica* are not only sulfated but acetated also [17,18]. The presence of acetyl groups in fucoidans were detected by NMR spectroscopy. The removal of acetyl groups from FeF via alkaline treatment gives FeFDA with a low molecular weight of 20 kDa. The characteristics of fucoidans under study are presented in Table 1.

Previous studies showed that α-l-fucans from *S. cichorioides* and *F. evanescens* exhibited antiviral activity against Hantaan virus [19], galactofucan from *S. japonica* had antiviral activity against avian influenza A (H5N1) virus infection [20] and in case of experimental tick-borne encephalitis. However, the α-l-fucans from *S. сichorioides* and *F. еvanescens* and GF from *C. costata* showed moderate activity in the same experiment [21].

### 2.2. Antiviral Activity

The investigated fucoidans at concentrations of up to 100 μg/mL had no cytotoxic effect on Jurkat and SC-1 cell line (data not shown).

#### 2.2.1. Model System Based on Lentiviral Vectors

A model system based on lentiviral vectors was used to study antiviral activity of fucoidans [16,22]. Pseudo-HIV-1 particles are recombinant lentiviruses based on HIV-1. They contain a set of HIV-1 enzymes and structural proteins, but pseudo-HIV-1 particles are not replication competent because they have the marker enhanced green fluorescent protein (eGFP) gene in their genome instead of viral genes. In fact, these pseudoviral particles are one-time disposable viruses. Pseudo-HIV-1 particles functional activity is provided by HIV-1 enzymes that catalyze synthesis of DNA provirus and its integration into the host cell genome.

Lentiviral transduction of the target cells by pseudo-HIV-1 particles leads to the marker gene expression that induces the fluorescence of the target cell. Transduced cells can be detected by flow cytometery. The compounds with anti-HIV-1 activity, which are inhibitors of HIV-1 life cycle, prevent the emergence of florescent cells in the population. 

Pseudo-HIV-1 particles can carry envelope proteins of HIV-1 or other enveloped viruses on their surface. Two types of pseudo-HIV-1 particles were obtained and subjected to study, namely particles that contain HIV-1 gp120+gp41 envelope protein and particles that contain G envelope protein from vesicular stomatitis virus (VSV).

#### 2.2.2. Inhibitor Activity of the Fucoidans Against Transduction of Jurkat Cells by Pseudo-HIV-1 Particles that Contain HIV-1 gp120+gp41 Envelope Protein

**Figure 1:**
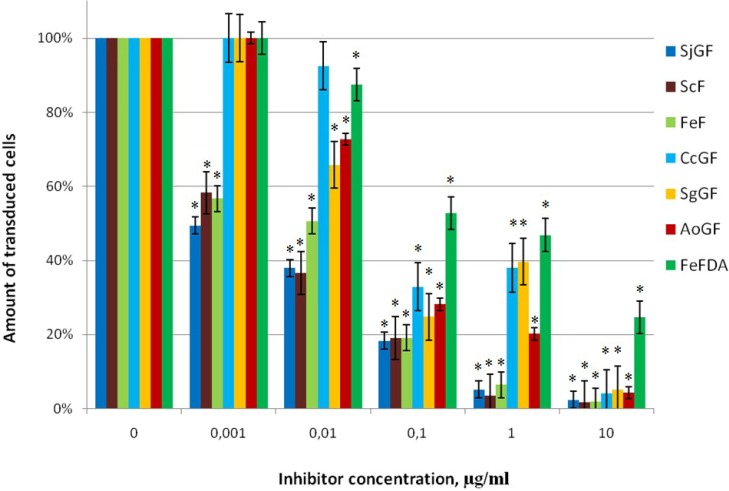
The action of fucoidans on transduction efficiency of pseudo-HIV-1 particles containing HIV-1 envelope protein gp120+gp41, Jurkat cells, 48 h. after transduction. The level of transduction is shown regarding the positive control, which was taken as 100%. Data points represent mean values ± standard deviation (SD) from three independent measurements, each performed in triplicates. * *p*-Value < 0.05.

Based on the chemical composition and MW, we determined the abilities of the above compounds to prevent lentiviral transduction of the Jurkat cells by pseudo-HIV-1 particles containing HIV-1 gp120+gp41 envelope protein. Fucoidans ScF, FeF, SgGF, AoGF, SjGF, CcGF, FeFDA added at a concentration of 0.001 up to 10 µg/mL in cell culture for 1 h before transduction were tested (Table 1, Figure 1). 

The fluorescence of cells were analyzed after 48 h as infection was made, this procedure is described in the Experimental Section.

All of studied fucoidans provide significant inhibition of lentiviral transduction of the Jurkat cells by pseudo-HIV-1 particles with HIV-1 gp120+gp41 envelope protein in concentration from 0.1 µg/mL (Figure 1). Fucoidans FeFDA and CcGF, with low Mw of 20 and 160 kDa, respectively, were not efficient at the concentration of 0.01 and 0.001 µg/mL. In contrast, fucan ScF and galactofucan SjGF with high Mw were of interest since their IC_50_ is 0.005 and 0.001 µg/mL, respectively. The variation of Mw amongst sulfated polysaccharides and their antiviral potency is shown in Table 2.

The important parameter for the antiviral activity is the degree of sulfation of polysaccharide. Recently, it was shown that the antiherpetic activity of several other families of polysaccharides, such as spirulan, agaran, fucan, xylomannan, and their desulfated and oversulfated derivatives, largely depends on the presence of sulfate groups [23,24,25,26,27]. According to Wituraw and de Clerc, the presence of the sulfate group is necessary for anti-HIV activity [8]. Sulfated polysaccharides can block viral entry into host cell by interfering with the attachment of virus to the cell-surface receptors. These macromolecules may exert their anti-HIV-1 activity by shielding off the positively charged amino acids present in the viral envelope glycoprotein gp120 [28,29]. The glycoprotein has several highly basic regions, which can interact with polyanionic regions of host-cell-surface molecules [30]. Queiroz *et al*. [13] suggested that not only the charges, but also the spatial disposition of the sulfate groups may be important in inhibiting reverse transcriptase activity of HIV. In general, sulfated polysaccharides with sulfate content higher than 20 mol% have a clear tendency of the antiviral activity demonstration [5]. In the present study, all investigated fucans and galactofucans were highly sulfated and showed strong inhibitory activity at 10 µg/mL. Although, highly sulfated galactofucans SgGF, AoGF and CcGF at a lower concentration had a lower inhibitory effect than the other fucans and SjGF studied here (Figure 1).

**Table 2:** Antiviral activity of investigated fucoidans. IC_50_ was counted using data obtained from three independent measurements, each performed in triplicates.

Seaweed source	Fraction	Mw, kDa	IC_50_ (Pseudo-HIV-1 particles containing envelope protein gp120+gp41 HIV-1) µg/mL *	IC_50_ (Pseudo-HIV-1 particles containing envelope protein VSV-G) µg/mL *	IC_50_ (Mo-MuLV) µg/mL **
*α-l-Fucans (F)*					
*S. cichorioides*	ScF	1160	0.0050 ± 0.0003	>100	0.0060 ± 0.0003
*F. evanescens*	FeF	620	0.0100 ± 0.0004	>100	0.0060 ± 0.0003
*F. evanescens*	FeFDA	20	0.52 ± 0.02	n.d.	4.50 ± 0.14
*Galactofucans (GF)*					
*S. gurjanovae*	SgGF	810	0.0450 ± 0.003	n.d.	0.250 ± 0.009
*Al. ochotensis*	AoGF	860	0.0550 ± 0.0009	n.d.	0.0540 ± 0.0025
*S. japonica*	SjGF	1800	0.00100 ± 0.00003	>100	0.0050 ± 0.0002
*C. costata*	CcGF	160	0.075 ± 0.005	n.d.	0.066 ± 0.003

IC_50_, inhibitor concentration, whereby contamination level desreases up to 50% against to check cells not to be acted by the inhibitor; n.d., not determined; * Jurkat cells; ** SC-1 cells.

It might be supposed that the acetyl groups may also contribute to the presence of polyanionic charges. Steric effects of acetyl groups change the structure of polyanionic regions and spatial accessibility of polysaccharide sulfate groups. Fucans ScF, FeF and FeFDA (the acetyl group removal from the acetylated FeF gave FeFDA) were used for comparing the effect of acetyl groups in the polysaccharide chain on the antiviral activity of fucoidans. However, the activity of these polysaccharides is not narrowly related to the presence of acetyl groups. Non-acetylated ScF showed the greatest antiviral activity. The activity of FeFDA decreased 50-fold compared to the FeF (IC_50_ changed from 0.01 to 0.52 µg/mL) (Table 2, Figure 1). However, it should be noted that removal of acetyl groups leads to the decrease of the molecular mass of polysaccharide. Among galactofucans SgGF, AoGF, SjGF, and CcGF, the greater antiviral activity showed acetylated galactofucan SjGF (IC_50_ = 0.001 µg/mL).

For all analyzed fucoidans, the dependence of antiviral activity on fucoidan molecular weight was studied. It was shown that galactofucans and fucans of higher molecular weight showed greater antiviral activity (Table 2). These results are in agreement with the data of other authors, which showed that of sulfated polysaccharides, such as carragenans, fucans demonstrated more activity against type-2 Herpes simplex than higher their molecular weight [8,31,32]. 

We can suppose that suppression of transduction of Jurkat cells by pseudo-HIV-1 particles that contain envelope protein HIV-1 is influenced by the molecular mass of fucoidan, more so than the degree of acetylation or sulfation, or fucoidan structural peculiarities.

#### 2.2.3. Inhibitor Activity of the Fucoidans Against Transduction of Jurkat Cells by Pseudo-HIV-1 Particles that Contain G Envelope Protein from VSV

**Figure 2:**
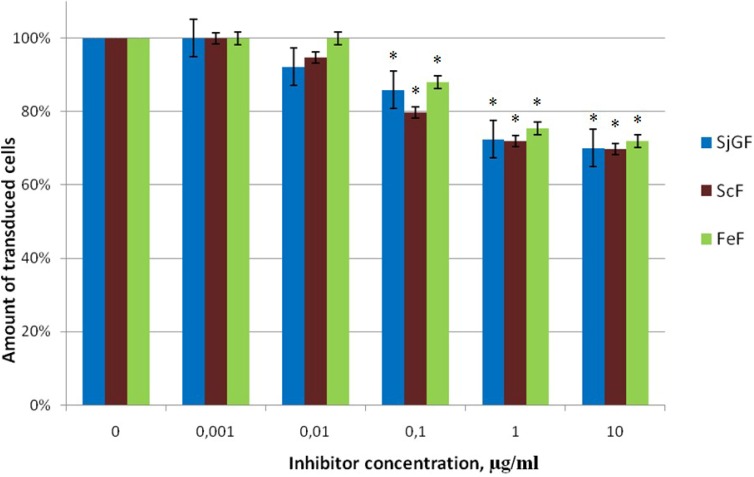
The action of fucoidans on transduction efficiency of pseudo-HIV-1 particles containing envelope protein VSV-G, Jurkat cells, 48 h after transduction. The level of transduction is shown regarding the positive control, which was taken as 100%. Data points represent mean values ± SD from three independent measurements, each performed in triplicates. * *p*-Value < 0.05.

At the next stage of the work, the ability of the fucoidans to prevent transduction of the Jurkat cells by pseudo-HIV-1 particles that contain VSV-G envelope protein was studied. The choice of envelope protein was based on the fact that these viruses have different mechanisms of cell penetration. Heparan sulfate serves as a primary cell receptor for the non-specific binding of HIV-1virus, while VSV penetrates into the cell by endocytosis mediated by the contact of envelope G protein with phospholipids of the cell membrane, unlike HIV-1 [33]. High molecular weight fucans, ScF, FeF and galactofucan SjGF, that effectively inhibited infection with HIV-1, were chosen for this experiment. The amount of transduced cells was decreased not more than of 20%–25% from control. So, all investigated fucoidans did not provide strong inhibition of antiviral activity against pseudo-HIV-1 particles with the envelope protein G of VSV in concentrations from 0.1 µg/mL (Figure 2, Table 2). 

Earlier, the fucoidans were shown to inhibit the HIV reverse transcriptase activity *in vitro* [13]. Our data illustrate the absence of this effect in our system based on lentiviral vectors. We suppose that fucoidans do not penetrate into target cells and have no effect on the reverse transcriptase as it was shown with isolated enzyme *in vitro*. 

We indicated that fucoidans do not prevent the transduction of marker eGFP gene by the pseudo-HIV-1 particles containing VSV-G envelope protein, this data indicate specific action of tested inhibitors against HIV-1.

#### 2.2.4. Inhibitor Activity of the Fucoidans Against Mo-MuLV Infection of SC-1 Cells

A model system based on a replication competent Moloney murine leukemia virus (Mo-MuLV) was used to study fucoidans ability to prevent virus infection extension in SC-1 cells. The Mo-MuLV used in our system is a simple retrovirus, which, like HIV-1, interacts with heparan sulfate as a primary receptor [15]. We used the recombinant Mo-MuLV retrovirus, with enhanced green fluorescent protein (eGFP) gene integrated into the 3′-end region of genome.

**Figure 3:**
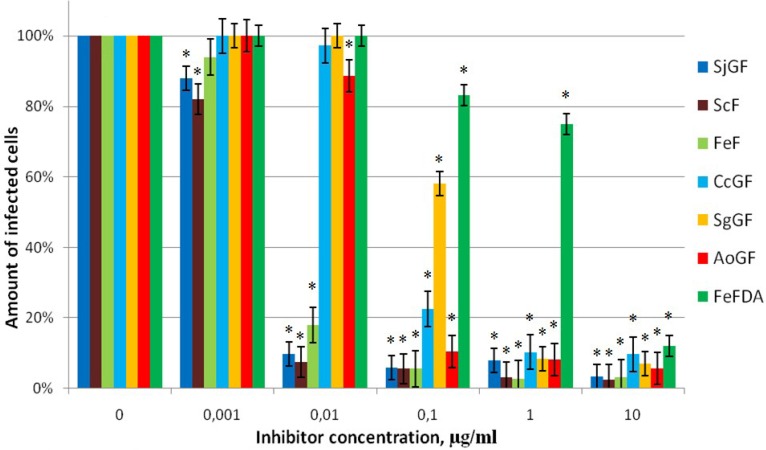
The action of fucoidans on efficiency of Mo-MuLV infection, SC-1 cells, 48 h after infection. The level of infection is shown regarding the positive control, which was taken as 100%. Data points represent mean values ± SD from three independent measurements, each performed in triplicates. * *p*-Value < 0.05.

The cells were treated with each investigated polysaccharide at a concentration from 0.001 to 10 µg/mL. Figure 3 shows dose response curves of examined compounds. 

The most effective inhibitors appeared to be high molecular weight fucoidans—fucans ScF (IC_50_ = 0.006 µg/mL), FeF (IC_50_ = 0.006 µg/mL) and galactofucan SjGF (IC_50_ = 0.005 µg/mL). The low Mw FeFDA was less effective (IC_50_ = 4.5 µg/mL). The antiviral activity of the preparation FeFDA decreased almost 500 times in comparison to FeF (Table 2). The action of other investigating galactofucans was effective and comparable to each other. These results correlate with the data obtained using pseudo-lentivirus particles contain HIV-1 gp120+gp41 envelope protein (Table 2). Therefore, it can be concluded that the antiviral effect of fucoidans is specific to retroviruses that use heparan sulfate as the primary cell receptor.

## 3. Experimental Section

### 3.1. Biological Material

Brown algae *Saccharina cichorioides* (two-year cycles of development) and *Costaria costata* were collected in July 2008 in the Troitsa Bay (Sea of Japan) at the Sea Experimental Station of Pacific Institute of Bioorganic Chemistry (PIBOC), Far-East Branch, Russian Academy of Sciences (Primorsky region of Russia). Brown algae *Fucus evanescens* and *Saccharina gurjanovae* were collected from the littoral at the west coast of the Iturup Island of the Sea of Okhotsk (Kuril Islands) in July 2011 and at the coast of the Island Big Shantar (Sea of Okhotsk) in July 2009, respectively. Brown algae *Alaria ochotensis* and *Saccharina japonica* were collected from natural habitats of Sakhalin Island (Okhotsk Sea) and Kunashir Island (Pacific Coast), respectively, in August 2008.

The algae species were identified by A. Skriptsova (A.V. Zhirmunsky Institute of Marine Biology FEB RAS, Russia).

### 3.2. Isolation and Determination of the Fucoidan Structure

#### 3.2.1. Extraction of Water-Soluble Polysaccharide from the Brown Algae

The fucoidans used in the present work are listed in Table 1. The procedure of the fucoidans isolation from *S. cichorioides* has been described earlier [34]. The procedure includes the following stages: extraction of the dry defatted algal biomass with a dilute solution of HCl (pH 2.0–2.3), dialyzation and lyophilization or precipitation of polysaccharides with ethanol and an ion-exchange chromatography of the native polysaccharide performed to obtain the most sulfated fraction ScF. The same procedure was used to obtain fucoidans FeF from *F. evanescens* [34], SgGF from *S. gurjanovae* [18], AoGF from *Al. ochotensis* and SjGF from *S. japonica* [35] and CcGF from *C. costata* [36].

The types of glycosidic linkage between fucose residues of fucoidans of ScF [37], FeF [38], SgGF [18], AoGF (unpublished data), SjGF [39] and CcGF [40] were described before.

#### 3.2.2. Analysis of Monosaccharide Composition

The monosaccharide compositions of fucoidans were determined as described previously [31]. The monosaccharide composition of the polysaccharides after acidic hydrolysis was determined on a Biotronik IC-5000 carbohydrate analyzer (Germany) using a Shim-pack ISA-07/S2504 (0.4 × 25 cm) column. Elution was performed with a potassium borate buffer at an elution rate of 0.6 mL/min. Detection was carried out by the bicinchoninate method and integration on a Shimadzu C-R2 AX system. Monosaccharides (Rha, Man, Fuc, Gal, Xyl, and Glc) were used as standards.

#### 3.2.3. Deacetylating of the Fucoidan

An alkaline treatment was used to remove acetyl groups from FeF giving FeFDA. Native polysaccharide FeF (500 mg) was dissolved in water (10 mL), than concentrated aqueous NH_4_OH (10 mL) was added to the solution. This mixture was kept at 60 °C for 4 h. A solution of polysaccharide was dialyzed by ultrafiltration (1 kDa cutoff) and lyophilized. 

#### 3.2.4. Sulfate Group Content

The sulfate group content was determined by the turbidimetric method after hydrolysis of the corresponding fractions with HCl (1 N) [41].

#### 3.2.5. Anion-Exchange Chromatography

Anion-Exchange Chromatography: polysaccharide solutions made in 0.04 N HCl (1 g/20 mL) were applied onto a DEAE-cellulose column (Cl-form, 3 × 14 cm) equilibrated with 0.04 N HCl. Sulfated polysaccharides were successively eluted with liner gradient of 250 mL H2O/250 mL NaCl (2 M). The fractions were dialyzed and lyophilized to obtain the polysaccharide fractions.

#### 3.2.6. Determination of Molecular Weight

The molecular weights (MW) of polysaccharides were determined by HPLC in a Shimadzu LC-20A instrument with a RID-10A refractometric detector. Polysaccharides were separated over successively connected columns of Shodex Asahipak GS-520 HQ and GS-620 HQ (7.5 mm × 300 mm) at 50 °C with elution by H2O (0.8 mL/min). Columns were calibrated using standard pullulans with MWs ranged from 180 to 667 kDa (Polymer Laboratories, Amherst, MA, USA) and blue dextran (Amersham, Uppsala, Sweden).

#### 3.2.7. NMR Spectroscopy

NMR spectra for solutions of the fucoidan in D_2_O were obtained on a Bruker Avance DPX-500 NMR spectrometer with a working frequency of 75.5 MHz at 60 °C.

### 3.3. Biological Assays

#### 3.3.1. Cell Culture

The following cell lines were used in this study: 

HEK293 (human embryonic kidney cells), SC-1 (mouse embryonic fibroblasts) were cultivated in DMEM containing 10% fetal calf serum (FCS), 4 mM of l-glutamine, 100 U/mL of penicillin, and 100 μg/mL of streptomycin.

Jurkat (human T-lymphoblastic leukemia) were cultivated in RPMI-1640 containing 20% FCS, 4 mM of l-glutamine, 100 U/mL of penicillin, and 100 μg/mL of streptomycin. 

The cells were grown at 37 °C in humid air containing 5% of CO_2_. 

#### 3.3.2. Generation of Jurkat Clone 4X4#3

##### Antibodies and FACS Analysis

Cells were stained with monoclonal antibodies against CD4 (#SK3), CD184 (#12G5) and CD195 (#2D7) or corresponding isotype controls (all BD, Heidelberg, Germany) according to the manufacturers’ instructions. FACS analysis was performed on a FACS CantoII (BD).

To establish S2-compatible viral infection assays, various human T-cell lines were screened for expression of CD4 and HIV co-receptors CXCR4 and CCR5 by FACS analysis. Among three different Jurkat cell lines tested, one cell line (Jurkat JMP, kindly provided by Andreas Guse, University Medical Center Hamburg-Eppendorf, Hamburg, Germany) showed partial expression of CD4 in more than 50% of the cells in conjunction with CXCR4 (data not shown). To obtain cells with homogenous expression of both receptors, cells showing the highest expression of both CXCR4 and CD4 were isolated via fluorescence-activated cell sorting on a FACS ARIA (BD). Sorted cells were seeded as single cell cultures into 96 well plates, expanded for 4 weeks and tested for CD4 and CXCR4 expression. One clone (4X4#3), which was highly positive for CD4 and CXCR4 (>99.5%) and showed stable receptor expression for several months in culture, was selected as target for subsequent infections.

#### 3.3.3. Obtaining of Pseudo-HIV-1 Particles

HEK293 cells seeded in Petri dishes with a diameter of 100 mm in the amount of 3.0–3.5 × 10^6^ cells per dish 12–14 h prior to the transfection were used as packaging cells, in which the assembly of recombinant pseudo-HIV-1 particles occurs. DNA of the lentiviral vector containing the marker eGFP gene and the plasmids directing the synthesis of the proteins that are required for the formation of pseudo-HIV-1 particles were introduced into HEK293 cells via calcium phosphate transfection. The pseudo-HIV-1 particles were collected 24 h following transfection with a 12 h interval [16].

The pseudo-HIV-1 particles were titrated on Jurkat cells seeded to 24-well plates 24 h prior to transduction. The level of cell fluorescence was measured on Beckman Coulter Epics 4XL flow cytometer (USA) 48 h following the transduction.

The pseudo-HIV-1 particles titer was calculated using the formula T = NP/V, where N is the amount of seeded cells, P is the share of the transduced cells in the population, V is the amount of the added supernatant containing pseudo-HIV-1 particles (mL), and T is titer. The samples with titer of 5 × 10^5^–5 × 10^6^ were used in this study. 

#### 3.3.4. Obtaining Replication Competent Virus Particles

The assembly of replication competent retrovirus particles and their secretion into the culture medium was carried out in SC-1 cells, which were seeded on a Petri dish 100 mm in diameter with (3.0–3.5) × 10^6^ cells per plate 12–14 h before transfection.

Retroviral vector containing the full length genome of Mo-MuLV, as well as the GFP marker gene, was introduced into SC-1 cells by Lipofectamine 2000 transfection. Replication competent retroviral particles were collected 48 h after transfection at an interval of 10–12 h. The virus was titrated on SC-1 cells seeded into 24 well plates (3 × 10^5^ cells per well) 14–16 h before infection as described above for Jurkat cells. 

#### 3.3.5. Cytotoxicity Assay

Cytotoxicity of preparations for uninfected Jurkat and SC-1 cells was determined on changes in morphology and the number of viable cells using Trypan blue staining solution (Invitrogene Corp., Carlsbad, CA, USA). For this purpose, the preparation of polysaccharide up to concentration of 10 and 100 µg/mL was added in the medium. After 48 h, Jurkat cells were resuspended in the medium and stained with 0.4% solution of Trypan blue for 5 min. SC-1 cells were removed by trypsin, resuspended in the medium and stained with 0.4% Trypan blue solution for 5 min. Then, the number of viable (unstained) and nonviable (stained) cells were counted in a Neubauer chamber. The number of living cells in the population was estimated by the number of unstained cells (as a percentage of the total).

### 3.4. Antiviral Activity Assay

Antiviral activity of fucoidans was determined on Jurkat cell line to which different amounts of analyzed preparations (20 mL per well) were added before lentiviral transduction by pseudo-HIV-1 particles and on SC-1 cell line to which the same amounts of analyzed preparations were added 1 h before Mo-MuLV infection. The number of fluorescent cells was counted on a flow cytometer 48 h after transduction. Fucoidans in concentrations of 0.01, 0.1, 1.0, 10.0, and 100 μg/mL per well were used.

Unpaired Student’s *t*-test was performed to evaluate significance of data groups; GraphPad t test calculator was used. The difference was considered statistically significant at *p* < 0.05. 

## 4. Conclusions

It can be concluded that antiviral activity of investigated fucoidans depends on the type of virus, more precise on the envelope protein species. The inhibitory activity of fucoidans is specific against viruses that use heparan sulfate as the primary cell receptor. The investigated fucoidans suppressed transduction of Jurkat cells by pseudo-HIV-1 particles that contain envelope protein HIV-1 regardless to structure of carbohydrate backbone. The data obtained allows us to consider all investigated natural high molecular weight fucoidans as potential anti-HIV agents, because their efficiency against lentiviral transduction was demonstrated at low concentrations (0.001–0.05 µg/mL). High molecular weight 1,3-α-l-fucan ScF and galactofucan SjGF had the best inhibiting effect. Investigation of these fucoidans’ antiviral activity will be continued.
